# Pubertal delay in children and adolescents with sickle cell disease in eastern Uganda: a cross-sectional study

**DOI:** 10.21203/rs.3.rs-10960024/v1

**Published:** 2026-10-19

**Authors:** Joanitah Nampijja, Kevin Otoro, Jimmy Patrick Alunyo, David Mukunya, Sarah Kiguli, Crispus Tegu, Lisa Rynn, Simon Odoch

## Abstract

Background Pubertal delay is a neglected complication of sickle cell disease (SCD), particularly in sub-Saharan Africa where disease burden is high and survival into adolescence is increasing. In these setting, we estimated the prevalence and correlates of pubertal delay among children and adolescents with SCD in eastern Uganda. Methods We conducted a hospital-based cross-sectional study among patients aged 8–19 years with confirmed SCD attending Mbale Regional Referral Hospital, Uganda. Pubertal development was assessed using Tanner staging and age-specific criteria for delayed puberty. Sociodemographic, nutritional, anthropometric, clinical, and treatment-related data were collected through structured interviews, medical history, and physical examination. Factors associated with pubertal delay were examined using multivariable ordinal logistic regression. Results Of 407 participants, 234 (57.5%) were female. Overall, 261 (64.1%) had delayed pubertal development, with higher prevalence among girls than boys (166 [70.9%] of 234 vs 95 [54.9%] of 173). Mean age at menarche was 13.15 years (SD 1.65). In adjusted analysis, male sex (aOR 0.28, 95% CI [0.16–0.47], p < 0.001), tall stature (aOR 0.39, 95% CI [0.17–0.90], p = 0.027), and intake of at least four fruit varieties per day (aOR 0.30, 95% CI [0.119–0.755], p = 0.011) were associated with lower odds of pubertal delay. Treatment status was associated with higher odds of pubertal delay (aOR 6.21, 95% CI [1.24-31.0], p = 0.026). Conclusion Pubertal delay was highly prevalent among children and adolescents with SCD in eastern Uganda. Routine pubertal, growth, and nutritional assessment should be integrated into comprehensive SCD care.

